# Evaluation of drug prescription pattern using World Health Organization prescribing indicators in public health facilities found in Ethiopia: systematic reviews and meta-analysis

**DOI:** 10.1186/s40545-021-00313-y

**Published:** 2021-03-19

**Authors:** Bereket Bahiru Tefera, Melese Getachew, Bekalu Kebede

**Affiliations:** Department of Pharmacy, Debre Markos University, College of Health Science, Po Box 269, Debre Markos, Ethiopia

**Keywords:** Drug use evaluation, Prescribing indicator, World Health Organization, Literature review, Drug use patter

## Abstract

**Background:**

Drug use evaluation is a structured, methodological, and criteria-based drug assessment system that helps to evaluate the actual trend of drug use in a particular setting. If drug prescription practices are inappropriate, need to examine the patterns of drug use is necessary to change prescribing patterns accordingly. Therefore, this review aimed to determine the drug prescription pattern in public health facilities found in Ethiopia using prescribing indicators developed by the World Health Organization.

**Methods:**

This review was conducted as per the PRISMA (Preferred Reporting Items for Systematic Reviews and Meta-Analyses) guideline. Extensive searching to identify articles was conducted in PubMed, Medline, Web of Science, Research Gate, Africa Journal of Online, and Google scholar. Finally, 10 eligible articles were selected for analysis based on inclusion and exclusion criteria. The median value, as well as the 25th and 75th percentiles for each WHO prescribing indicator, were computed.

**Result:**

The pooled median value of WHO prescribing indicators was reported as follows: the average number of drugs prescribed per encounter = 2.14 (IQR 1.79–2.52), the percentage of encounters with antibiotics prescribed = 43.46% (IQR 30.01–58.67), the percentage of encounters with an injection prescribed = 13.20% (6.47–40.7), percentage of drugs prescribed by generic name = 93.49% (89.13–97.96), and the percentage of medicines prescribed from essential medicines list = 92.54% (85.10–97.7). The forest plots determined for each prescribing indicator indicated that there is a high degree of heterogeneity across articles.

**Conclusion:**

All of the prescribing indicators were not consistent with the standard values recommended by the World Health Organization. Therefore, public health facilities should take appropriate measures for improving the prescription patterns as per the recommendation set by the World Health Organization.

**Supplementary Information:**

The online version contains supplementary material available at 10.1186/s40545-021-00313-y.

## Background

Drug use evaluation is a structured, methodological, and criteria-based drug assessment system that helps to evaluate the actual trend of drug use in a particular setting. It is a system of collecting information to identify issues related to drug use and, eventually, to take steps to address the identified problem. Evaluation of drug use has a significant role to play in encouraging the rational use of pharmaceutical drugs and effective prescribing patterns [1, 2]. Reasonable drug use (RDU) includes proper prescribing; drug dispensing; and patient use for diagnosis, prevention, and disease treatment [3]. The rational use of medicines allows patients to obtain drugs relevant to their clinical indication at the lowest cost to them in doses that fulfill individual requirements over a reasonable period [4]. Irrational drug use exists in all parts of the world and the typical types of irrational drug use include insufficient dose, poly-pharmacy, improper use of antimicrobial agents; overuse of injections when oral dosage forms are more applicable; and failure to prescribe according to the standard therapy guideline (STG) [5].

As reported by the World Health Organization (WHO), more than half of all medicines in the world are inappropriately prescribed in developing countries, where monitoring and evaluation of drug utilization are at an embryonic stage [6]. In addition, nearly, one-third of the world's population lacks access to essential medicines [1]. Irrational drug use will cause excessive community healthcare demand, and inevitably there will be medication stock-outs and deterioration of patient trust in the quality of health care service [7]. Inappropriate prescription practices lead to ineffective and dangerous treatment, exacerbation or prolongation of the patient's disease, and exaggerated costs. If drug prescription practices are inappropriate, the need to examine the patterns of drug use is necessary to change prescribing patterns accordingly [8]. For this purpose, several well-recognized survey approaches have been developed and one of them is an assessment based on WHO drug use indicators. These indicators are widely recognized as a global standard for health facilities' drug patter assessment [5]. Various studies have been undertaken to determine the prescribing pattern of public health facilities in Ethiopia. However, there has been no thorough systematic review or meta-analysis of these studies to provide an overall picture of the pattern of drug use in the country. Therefore, this review aimed to determine the drug prescription pattern in public health facilities found in Ethiopia using prescribing indicators developed by the World Health Organization.

## Methods

### The review protocol

The identification, eligibility screening, and selection of articles for this review were conducted as per the Preferred Reporting Items for Systematic Reviews and Meta-Analyses (PRISMA) flow diagram. Besides, this review followed the PRISMA checklist for conducting the review [9].

### Articles searching strategies

Article searching was conducted in different genuine databases including PubMed, Medline, Web of Science, Research Gate, Africa Journal of online, and Google scholar. Articles were also searched manually using the reference lists cited by already identified studies. The keywords used for searching literatures include the World Health Organization, public health facilities, health facilities, drug use patterns, rational drug use, prescribing indicators, prescribing patterns, drug use indicators, prescribing evaluation, and Ethiopia. Besides, Boolean operators (AND, OR), and truncation were used properly for identifying articles to be include in this review. Gray pieces of literature were also retrieved from the websites of different universities and other organizations of Ethiopia. The search was conducted from 1 to 30 March 2020.

### Screening of eligible articles

The study area and setting, study design, study objectives, study population, sample size and sampling techniques used, methods used for data collection, and statistical analysis were thoroughly evaluated to verify the eligibility of those articles. After a thorough evaluation of the articles, these met the inclusion and exclusion criteria were selected for the analysis.

### Inclusion and exclusion criteria of studies

This review included articles conducted to assess drug use patter at public health facilities located in Ethiopia and published between January 2015 and June 2020. This review aimed to assess the recent status of drug use patterns in public health facilities, so this timeline was optimal for bringing the status of the intended variables. Besides, articles included in this review were used random sampling techniques to select their samples for data collection. And only articles reported all the WHO drug prescribing indicators were included in this review. In the case of duplicated publications, the version released earlier or the other with full details was picked. The authors evaluated the accuracy of the calculation of each article during the determination of the prescription indicators, and the articles with errors of calculation were omitted from this review.

### Evaluation of articles quality and publication bias

The quality of each article was measured using a 14-point points checklist adapted from previous literature [6, 10]. A one-point score was awarded if the study met each criterion. If the analysis did not meet the criteria, it received null. The quality rating was expressed as a percentage. An article is considered to be of 'high quality' if it scores greater than and equal to 70% of the total score. A score of 69–51% was considered "moderate quality" and "poor quality" was graded at a score of less than or equal to 50%. This evaluation was conducted to assess the internal and external validity of articles and to decrease the risk of biases. The mean score of two authors was taken for the final decision and articles with a score less than to 50% were excluded for analysis. In this review, there was not a formal assessment of publication bias could be performed, because the conventional approaches such as funnel plots and tests for asymmetry are considered unsuitable for proportion studies [11].

### Extraction of data from articles

A data extraction tool, with Microsoft Excel 2016 ®, was prepared by the authors to collect the data regarding the variables being analyzed, such as the average number of medicines per encounter; the percentage of antibiotic prescriptions; the percentage of injection prescriptions; the percentage of generic drugs prescribed; and percentage of prescribed drugs from essential drug list. Besides, the data related to the article characteristics, such as study location, authors, year of publication, study design, duration of data collection, type of health facility, number of health facilities, and number of prescriptions, were extracted. The approach adopted by this review was to assess each article as a single data point of equal weight without considering the number of prescriptions evaluated by each article to minimize the effects of larger sample size articles, as in other previous reviews [6].

### Statistical analysis

The pertinent data were extracted from selected articles using a tool prepared in Microsoft Excel. The pooled estimate of median value as well as the 25th and 75th percentiles, for each WHO prescribing indicator, were calculated using Microsoft Excel. The mean values of the prescribing indicators were not used in this review, because they would be excessively skewed by outsiders. Using Microsoft Excel 2016 ®, all statistical calculations were done and the results of each prescribing predictor were compared to the suggested WHO standard values. The data were exported to OpenMeta Analyst software and the heterogeneity across the articles was assessed by determining the I^2^ statistics using Der Simonian and Laird’s random-effects model at a 95% confidence level. The prescribing indicators used in this review to measure drug use patterns in health care facilities include; Average number of medicines per encounter; Percentage of antibiotic prescriptions; Percentage of injection prescriptions; Percentage of generic drugs prescribed; and Percentage of prescribed drugs from the essential drug list [12].

## Result

### Articles identification and retrieval process

**Fig. 1:**
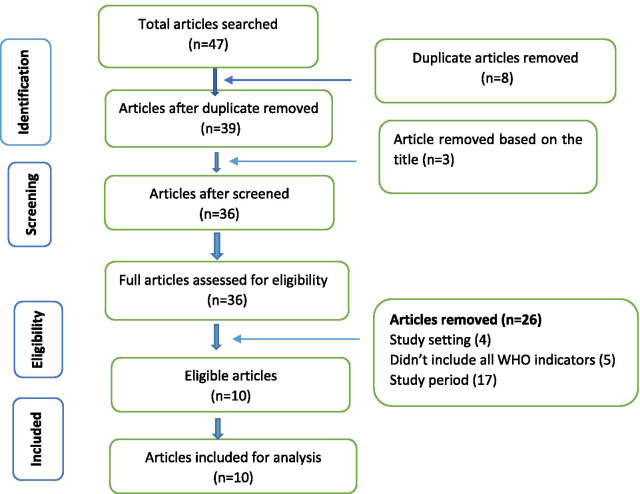
Articles identification and retrieval process

Initially, a total of 47 articles from all databases were identified. As shown in Fig. 1, first a total of 47 articles were identified. In addition, after thorough evaluation, based on inclusion and exclusion criteria, 10 articles were selected for analysis in this review.

### Characteristics of the articles included in the review

As indicated in Table 1, all articles covered in this review included a total of 12.130 prescriptions. A total of 39 public health facilities were included in the articles selected for analysis in this review. The majority of the articles selected for anlysis, 80% [8], collected data for the study duration of 1 year. On the other hand, one study collected data for the 6-month study period and another study also took 3 months of data. All studies were published after 2018, of which 50% [5], 30% [3], and 20% [2] were published in 2018, 2019, and 2020, respectively. Among articles included for analysis, only 50% of them utilized more than 600 sample sizes for assessing drug prescribing patterns.

**Table 1:** Characteristics of articles included in this review

Author Details	Year of Publication	Study area	Study design	Duration of Data collection	Type of health facility (s)	No. of health facilities	No. of prescriptions
Berhad and seyoum	2018	Addis Ababa city	Retrospective cross-sectional	February/1/2015–January/ 31/ 2016	Tikur Anbessa Specialized hospital	1	2000
Mishore et al	2020	Dire Dawa city	Retrospective cross-sectional	July/20/2018–August 19/ 2018	Dilchora Referral hospital	1	344
Yimenu et al	2019	Gondar city	Retrospective cross-sectional	March/1/2018–March/30/ 2019	Gondar Referral hospital	1	600
Wubetu et al	2018	Finote Selam town and Motta town	Retrospective cross-sectional	March/1/2015–February/ 29/2016	Finote Selam District hospital, and Motta district hospital	2	362
Gebramariam and Ahmed	2019	West Shoa Zone	Retrospective cross-sectional	January/1/2017–December /31/2017	Ambo referral hospital, Ambo General hospital, Gindeberet Primary Hospital, Gedo Primary Hospital, Jaldu Primary hospital, and Enchine Primary Hospital	7	2100
Assefa et al	2018	Adiss Ababa city	Retrospective cross-sectional	May/1/2015–October/31/2015	Tikur Anbessa Specialized hospital	1	384
Gashaw et al	2018	Harer region	Retrospective cross-sectional	January/1/2016–December/31/2016	Hiwot Fana Specialized Hospital, Federal Harar Police Hospital, Jugel Hospital, and Southeast Command III Hospital	4	2400
Bekele and Tadesse	2018	Dilla town	Retrospective cross-sectional	September/1/2016–August/31/2018	Dilla referral hospital	1	1440
Mamo and Alemu	2020	Dessie city	Retrospective cross-sectional	February/1/2019–May/31/2019	Dessie referral hospital	1	500
Wogayehu et.al	2019	Southern Ethiopia	Retrospective cross-sectional	January 2018 and December 2018		10	1000

### Quality appraisal of included articles

Among all the articles included in this review, 60% of them were classified as high quality (Additional file 1: Table S1). Besides, 30% and 10% of the articles were identified as medium and low quality, respectively. From all criteria used for evaluating the quality of the articles, WHO standards for the classification of drugs as injections; how to count drugs; and the classification of drugs as antibiotics were the criteria with the lowest quality levels with a percentage of 10%, 20%, and 30%, respectively.

### Outcome measures of the review

#### The pooled estimate of WHO prescribing indicators

Among the WHO prescribing indicators assessed in this review, the pooled median value of the “Average number of medicines prescribed per encounter” was 2.14 (IQR 2.52–1.79) (Table 2). Besides, the pooled median value of the “Percentage of encounters with antibiotics” reported with this review was 43.46% (IQR 58.67–30.01). This review also revealed that the computed pooled median value of the “Percentage of encounters with injection” was 13.20% (IQR 40.7–6.47). The pooled median value of the “Percentage of drugs prescribed by the generic name”, and the “Percentage of drugs from an essential drug list” was 93.49% (IQR 97.96–89.13), and 92.54% (IQR 97.7–85.10), respectively.

**Table 2:** Descriptive statistics of WHO prescribing indicators evaluated with this review

WHO indicators	Median (IQR)	WHO standard
Average number of drugs per encounter	2.14 (2.52–1.79)	< 2
Percentage of encounters with antibiotics	43.46% (58.67–30.01)	20%–26.8%
Percentage of encounters with injections	13.20% (40.7–6.47)	13.4%–21.1%
Percentage of drugs prescribed by generic name	93.49% (97.96–89.13)	100%
Percentage of drugs from essential drug list	92.54% (97.7–85.10)	100%

**Fig. 2:**
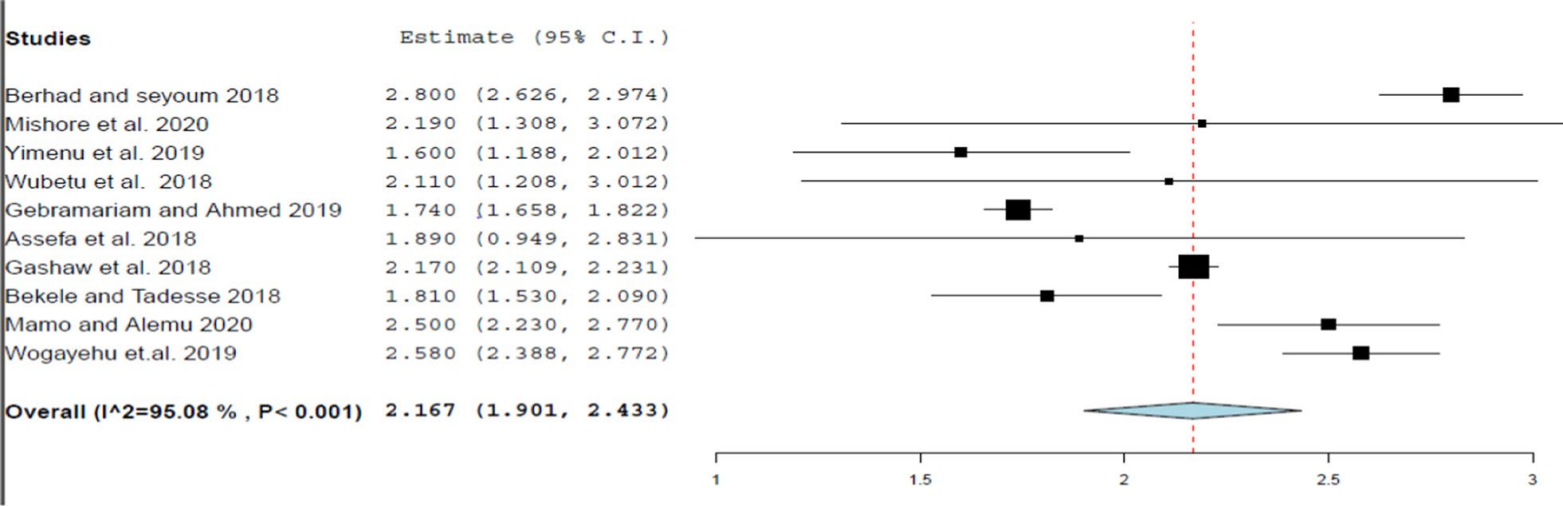
Forest plot of the average drugs prescribed per encounter

**Fig. 3:**
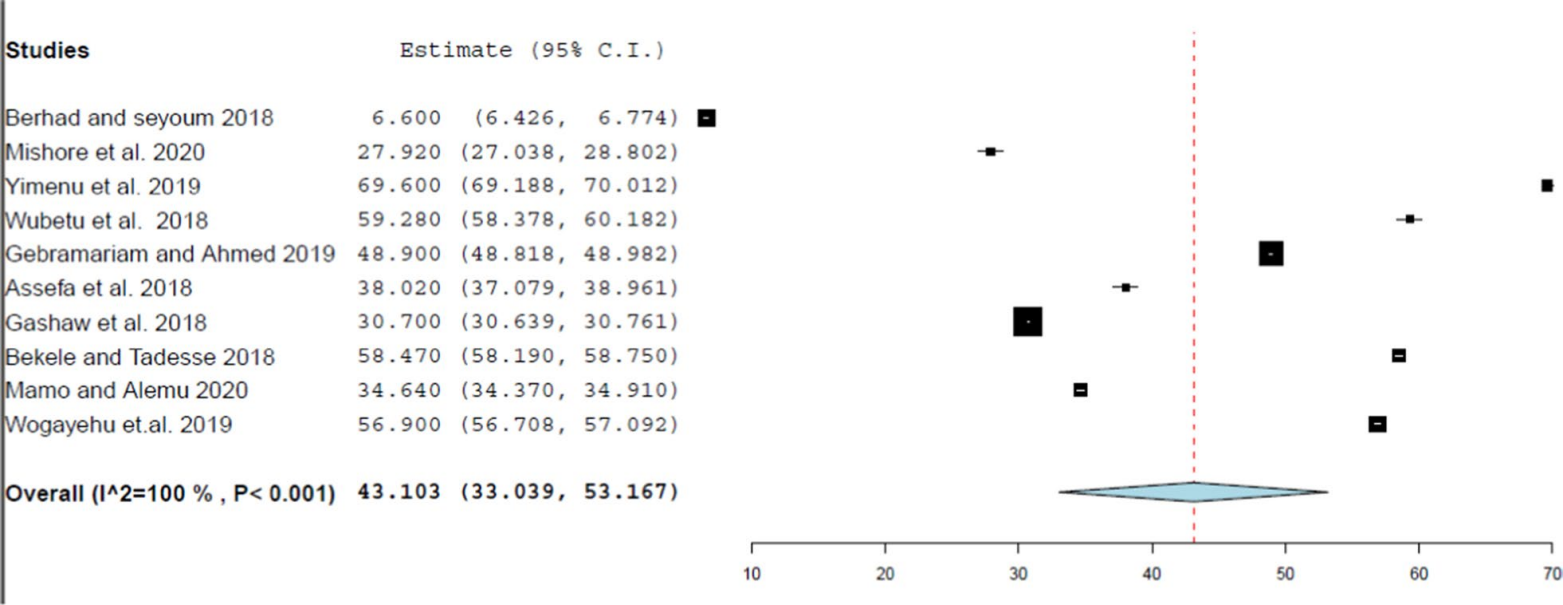
Forest plot of the pooled estimate of percentage encounter with antibiotics

**Fig. 4:**
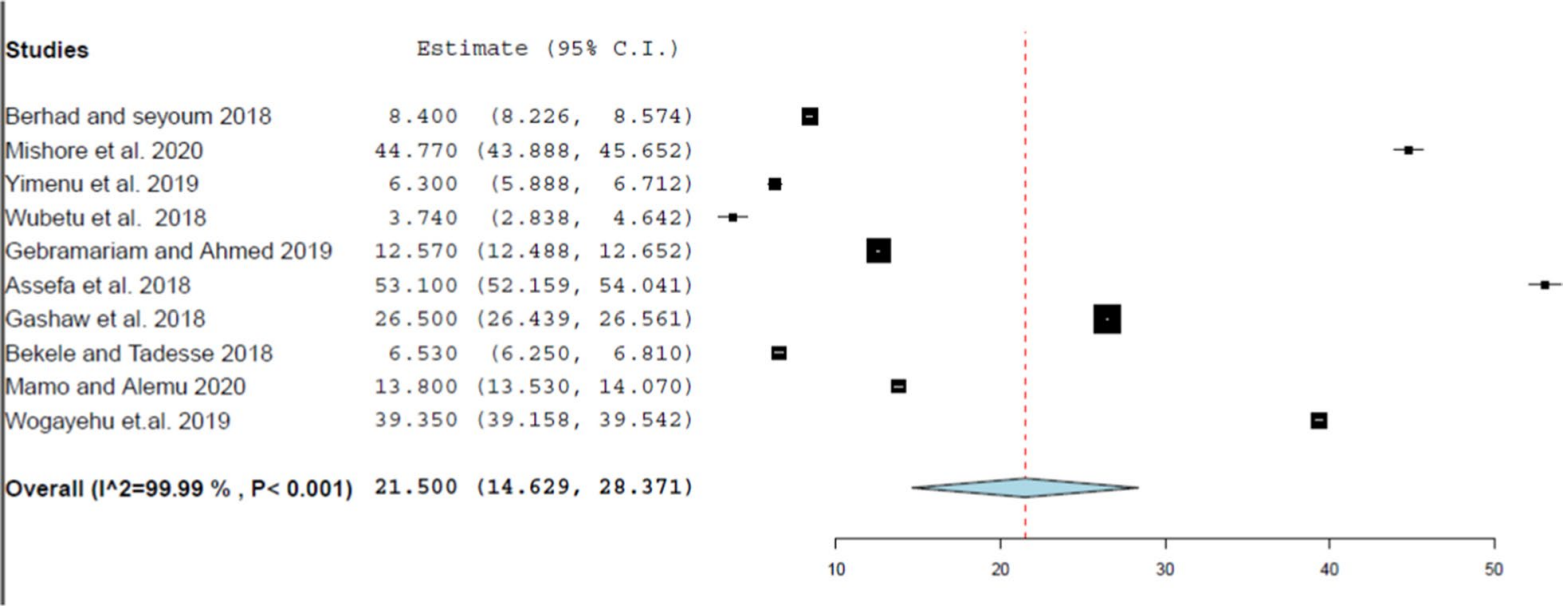
Forest plot of the pooled estimate of percentage encounter with injection

**Fig. 5:**
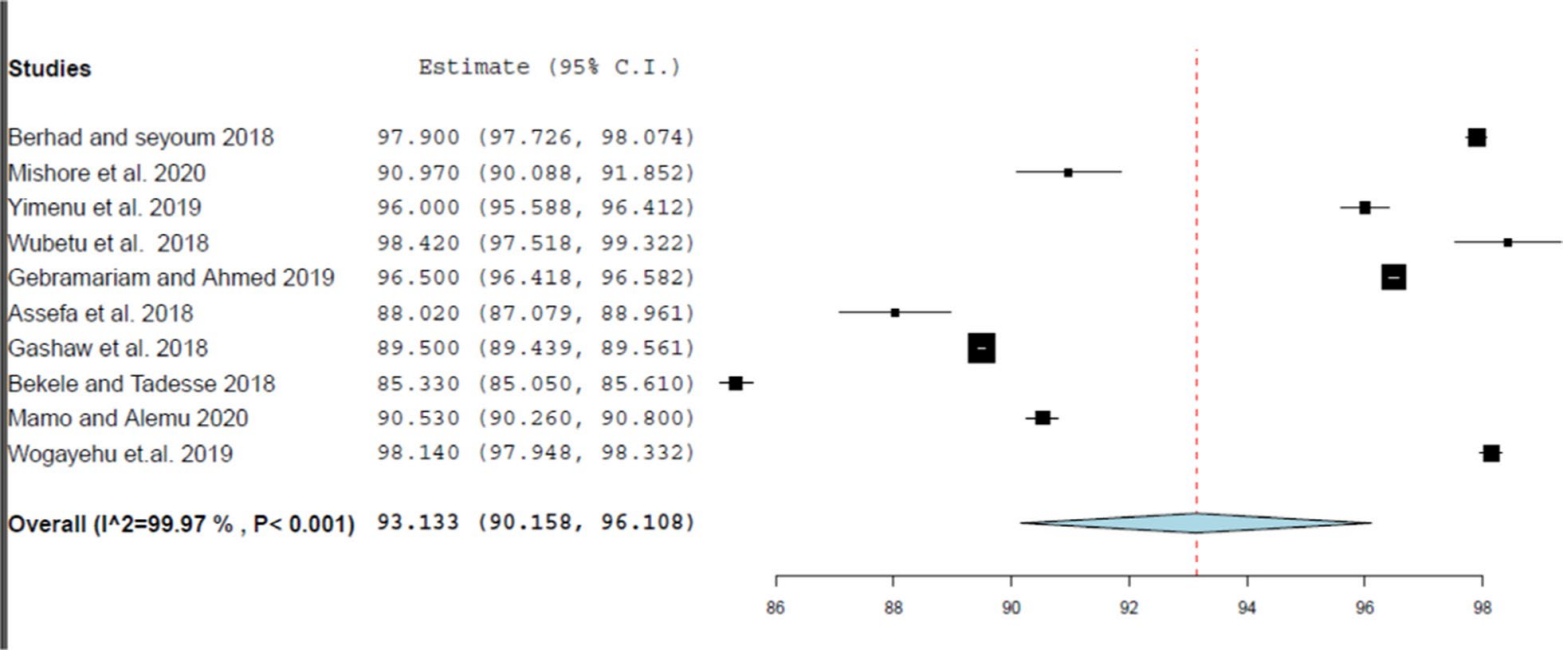
Forest plot of the pooled estimate of percentage drugs prescribed with generic names

**Fig. 6:**
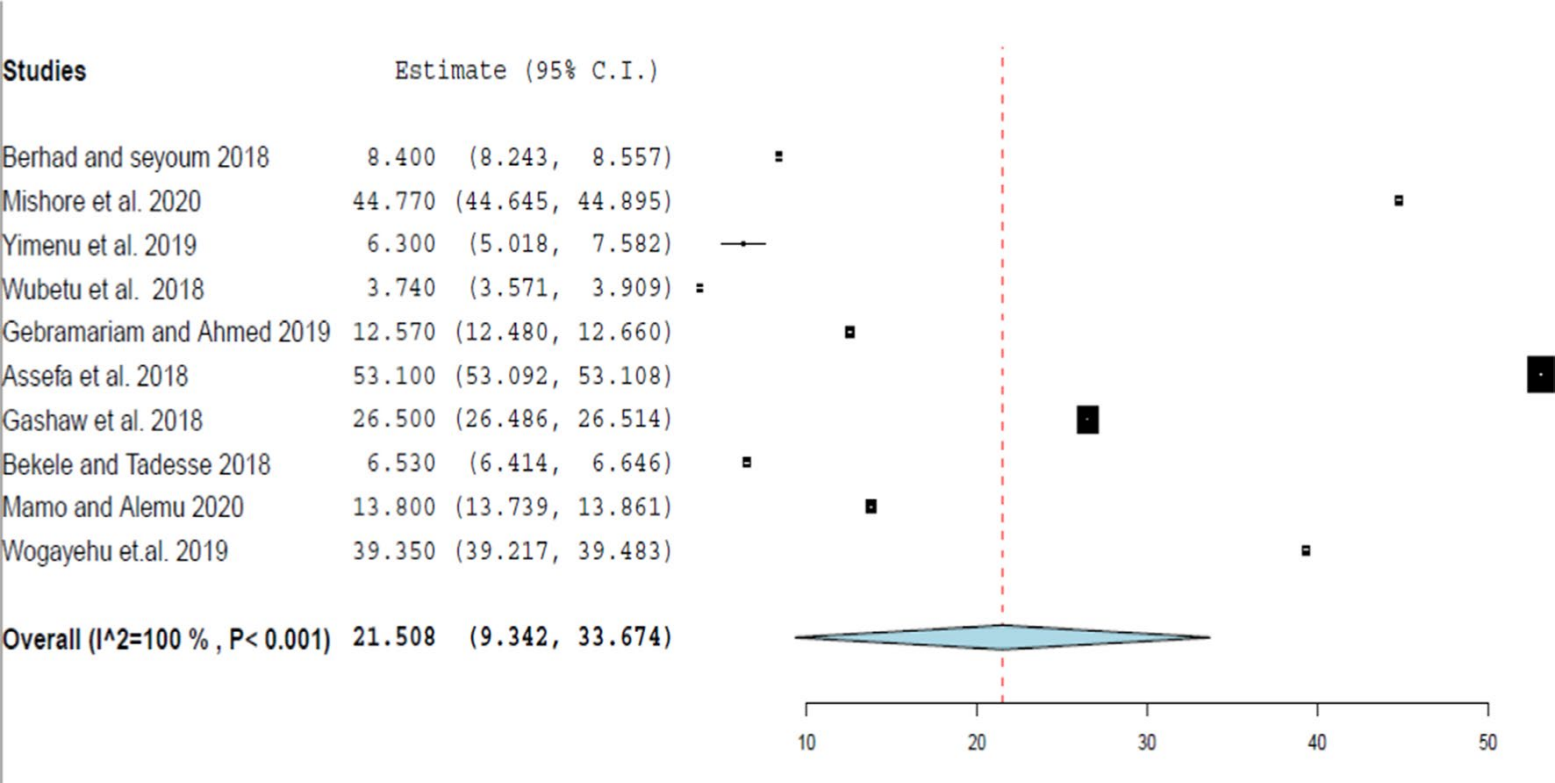
Forest plot of the pooled estimate of percentage drugs prescribed from essential drug lists

This review evaluated the heterogeneity across studies, for each WHO prescribing indicator, using I^2^ statistics using Der Simonian and Laird’s random-effects model at a 95% confidence level. As indicated in Figs. 2, 3, 4, 5, and 6 the forest plots determined for each prescribing indicator show that there is a high degree of heterogeneity across articles.

### Subgroup analyses of WHO prescribing indicators

We also conducted a subgroup analysis based on the sample size utilized by each article. The reference sample size was 600, which is the WHO recommended sample size for assessing rational drug use in healthcare facilities [13]. The articles with a sample size less than or equal to 600 were categorized in one group, whereas articles that conducted an assessment on sample size greater than 600 were clustered in the other group.

Subgroup analysis identified that the highest number of drugs per encounter was reported in the subgroup with a sample size greater than 600 with a median and IQR value of 2.22 (1.78–2.69) (Table 3). On the other hand, the subgroup with a sample size of less than or equal to 600 reported the highest percentage of encounters with antibiotics (45.89 (31.28–64.44). Similarly, the pooled estimate of the percentage of encounter with injections discovered in the subgroup with sample size less than or greater than 600 {with pooled median value and IQR of 24.34 (5.02–48.94)} was more than the other subgroup {with pooled median value and IQR of18.67 (7.47–32.92)}. However, subgroups with a sample size greater than 600 reported the highest pooled estimate of both the percentage of drugs prescribed with generic name and percentage of drugs from essential drug list with 93.47 (87.42–98.02) and 96.01 (91.66–99.65), respectively.

**Table 3:** Subgroup analysis of articles describing the WHO prescribing indicators separated based on the sample size

WHO indicators	Articles subgroup based on the sample size		WHO standard value
	Sample size ≤ 600	Sample size > 600	
	Median(IQR)	Median (IQR)	
Average number of drugs per encounter	2.06 (1.745–2.34)	2.22 (1.78–2.69)	< 2
Percentage of encounters with antibiotics	45.89 (31.28–64.44)	40.31 (18.65–57.69)	20%–26.8%
Percentage of encounters with injections	24.34 (5.02–48.94)	18.67 (7.47–32.92)	13.4%–21.1%
Percentage of drugs prescribed by generic name	92.79 (89.28–97.21)	93.47 (87.42–98.02)	100%
Percentage of drugs from essential drug list	90.53 (84.34–96.9)	96.01 (91.66–99.65)	100%

## Discussion

The findings of all WHO prescribing indicators reviewed in this systematic review were not consistent with the standard recommended by World Health Organization. However, among all prescribing indicators, the average number of drugs per encounter and percentage of encounters with injections were the indicators relatively close to the standard range recommended by WHO. Therefore, public health facilities in Ethiopia should improve the prescription pattern of all measures assessed by this review, especially the number of prescribed antibiotics in each prescription, prescribing drugs generic name, and prescribing drugs specified in the essential drug list prepared by them.

This review discovered that an average of 2.14 medicines have been prescribed per each prescription. Even though this finding is somewhat greater than but it is still very close to the standard value suggested by the WHO, which is less than 2 medicines per prescription [6]. This little disparity may be due to the fact that most parts of developing countries, especially African countries, are experiencing an epidemiological change that creates the disease burden of both communicable and chronic diseases [14]. Therefore, poly-pharmacy is also more obvious when healthcare professionals need to treat several diseases concurrently. The average number of medicines per prescription identified in this review was lower than the finding of a review done at primary health centers in the WHO Africa region (3.1) [6]. The discrepancy can be correlated with the fact that this review included articles that were done at all levels of the healthcare system (including hospitals, health centers, and clinics), while the other review was conducted only at primary healthcare centers. According to the subgroup analysis, the highest number of drugs per encounter was reported in the subgroup with a sample size greater than 600.

The median value of the percentage of encounters with prescribed antibiotics was 43.46%, which is almost twice the standard value recommended by the WHO (20%–26.8%) [15]. This over-prescription of antibiotics in Ethiopia maybe because the prevalence of various infectious diseases in Ethiopia is enormous [16]. Various studies also concluded that the excessive use of antibiotics and the lack of adherence to standard treatment guidelines substantially increased the prevalence of antibiotic resistance; therefore, this over-prescription of antibiotics may increase the antimicrobials resistance and ultimately lead to extended hospitalization and risk of death [17, 18]. Therefore, this implies that the risk of antimicrobial resistance, due to the over-prescription of antibiotics, is high in Ethiopia. Besides, this result was almost equivalent to a median percentage of 46.8% in a review conducted at primary health care centers within the WHO Africa region (36.2%) [6]. However, it was lower than the finding of another review conducted at low-and middle-income countries (52%) [20]. In addition, according to the subgroup analysis conducted in this review, the subgroup of articles with a sample size of less than or equal to 600 reported the highest pooled estimate of the percentage of encounters with antibiotics.

The pooled median value of the percentage of encounters with prescribed injections reported in this review was 13.20% and it is almost consistent with the range of the standard value recommended by WHO (13.4%–21.1%) [15]. This finding was also relatively lower than the review performed at primary health care centers in the WHO Africa region, which reported a 25% injection use rate [6]. In comparison, this result was relatively less than the findings of the review in Ethiopia with an injection average of 18.3% [12]. This difference may be associated with the fact that this review included articles conducted after 2015, while the other review included all studies conducted since the beginning of the 1990s, and during this period injection dosage form was relatively the most widely used in Ethiopia [21]. Additionally, the pooled estimate of the percentage of encounters with injections discovered in the subgroup with a sample size less than or greater than 600 was more than the other subgroup.

The computed median value of the percentage of prescriptions with the generic name reported by this review was 93.49%. This finding was somewhat lower than the standard value recommended by the WHO, which is 100%, but this disparity is not noteworthy [15]. The key advantage of the use of generic medicines is due to their low-priced nature, as they cannot be marketed at a price higher than the branded medicine, meaning that patients can adhere to their medicines as prescribed by the doctor [22–24]. However, this finding of this review was significantly higher than the generic prescribing rate reported by the review carried out at primary health centers in the WHO African region (60%) [6]. The subgroup analysis conducted in this review revealed that subgroups with a sample size greater than 600 reported the highest pooled estimate of the percentage of drugs prescribed with the generic name.

Finally, this review reported that the median value of the percentage of prescribed medicines adhering to the essential medicines list was 92.54%, which was somewhat lower than the standard value suggested by the WHO(100%) [15]. Compliance with the list of essential medicines is one of the key tools for a stable health care delivery system, as it ensures the availability and affordability of quality medicines at all care providers thereby promotes the rational use of medicines [25–27]. On the other hand, the verdict reported by this review somewhat exceeded the finding reported by a review conducted at primary health care centers within the WHO African Region (87.8%) [6]. Besides, the subgroup analysis of this review discovered that subgroups with a sample size greater than 600 reported the highest pooled estimate of the percentage of drugs prescribed from the essential drug list.

## Limitation

This systematic review has some limitations. Half of the articles included for analysis used a sample size less than 600 and it is not recommended by the WHO [13]. This review was based on the finding of indicator-based studies that are unable to determine whether the prescribed medicines were actually taken by patients or not. Each article included in this review was taken into account equally regardless of the number of prescriptions used for data collection, and the time variation when the data collection was done but these assumptions may affect the drug prescription patterns. Therefore, it would be more meaningful if the review treated each article differently based on the number of prescriptions used and the season when the data was collected. The core WHO prescribing indicators are usually used to evaluate the drug use trend at outpatient settings; therefore, this review does not give insight into the prescribing pattern in inpatient settings of public health facilities. Accordingly, this review recommended for future researchers who are interested in conducting studies in this field to take these shortcomings into account in their studies.

## Conclusion

This review demonstrated that the findings of all of the prescribing indicators were not consistent with the standard values suggested by the World Health Organization. However, among all prescribing indicators, the average number of drugs per encounter and percentage of encounters with injections were the indicators relatively close to the standard range recommended by WHO. Therefore, public health facilities in Ethiopia should improve the prescription pattern of all measures assessed by this review, especially the number of prescribed antibiotics in each prescription, prescribing drugs generic name, and prescribing drugs specified in the essential drug list prepared by them. Indeed, this review is based on a few studies conducted in Ethiopia, but it gave some insight into the need to improve the prescribing patterns in public health facilities found in Ethiopia. Therefore, public health facilities and stakeholders should devote their resources to making progress in the use of medications, as it plays a major role in maintaining community quality healthcare administrations.

## Supplementary Information

**Additional file 1: Table S1. **The checklist of the quality appraisal of articles.

## Abbreviations

EML: Essential Medicine List
IQR: Inter Quartile Range
PRISMA: Preferred Reporting Items for Systematic Review and Meta-Analysis
RDU: Rational Drug Use
STG: Standard Treatment Guidelines
