# Supplementary material for: Evaluation of drug prescription pattern using World Health Organization prescribing indicators in public health facilities found in Ethiopia: systematic reviews and meta-analysis
Source: J Pharm Policy Pract. 2021 Mar 19;14:31. doi: 10.1186/s40545-021-00313-y (PMC7980606; doi:10.1186/s40545-021-00313-y)
Supplement: Supplementary file 1 — Additional file 1: Table S1. The checklist of the quality appraisal of articles. [file 40545_2021_313_MOESM1_ESM.docx]

Table 2: The checklist of the quality appraisal of articles

| **CHECKLIST for quality appraisal** | **A1** | **A2** | **A3** | **A4** | **A5** | **A6** | **A7** | **A8** | **A9** | **A10** |
| --- | --- | --- | --- | --- | --- | --- | --- | --- | --- | --- |
| Clearly defined the objective of the study | 1 | 1 | 1 | 1 | 1 | 1 | 1 | 1 | 1 | 1 |
| Clearly defined the methods of the study design or data collection | 1 | 1 | 1 | 1 | 1 | 1 | 1 | 1 | 1 | 1 |
| Participants representing a general population of the patient (the study of prescribing indicators involved a mix of health problems) | 1 | 1 | 1 | 1 | 1 | 1 | 1 | 1 | 1 | 1 |
| The study used an adequate sample size (WHO suggests at least 600 gatherings) | 1 | 0 | 1 | 0 | 1 | 0 | 1 | 1 | 0 | 1 |
| The type of health facility (i.e. public or private) has been specified | 1 | 1 | 1 | 1 | 1 | 1 | 1 | 1 | 1 | 0 |
| The number of involved health facilities was specified | 1 | 1 | 1 | 1 | 1 | 1 | 1 | 1 | 1 | 1 |
| The age / gender of patients and other characteristics reported | 1 | 1 | 1 | 1 | 1 | 1 | 1 | 1 | 1 | 1 |
| The study explained how medicines were counted (WHO) suggested FDCs should be counted as one) | 0 | 0 | 1 | 0 | 1 | 0 | 0 | 0 | 0 | 0 |
| The study explored the drugs to be considered as antibiotics according to the classification WHO/ INRUD indicator | 0 | 0 | 1 | 0 | 1 | 0 | 0 | 1 | 0 | 0 |
| The reference of the list of essential medicine of (EML) used in the study was mentioned (researchers can use the WHO EML model, facility's EML or national EML as a reference guide) | 1 | 1 | 1 | 0 | 1 | 1 | 1 | 1 | 1 | 0 |
| The drugs known as injections were specified in the study (routine immunizations should not be counted as injections) | 0 | 0 | 0 | 0 | 1 | 0 | 0 | 0 | 0 | 0 |
| The statistical method used to evaluate the outcomes of the analysis was acceptable and fully described | 1 | 1 | 1 | 0 | 0 | 1 | 1 | 1 | 1 | 1 |
| The study described the way missing data and confounding factors were treated | 1 | 1 | 1 | 0 | 1 | 0 | 1 | 0 | 0 | 1 |
| The results of the study were adequately discussed. (e.g. where the conclusion important to the findings) | 1 | 1 | 1 | 0 | 1 | 0 | 1 | 0 | 1 | 1 |
| **PERCENTAGE (%)** | **78.57** | **71.43** | **92.86** | **50** | **92.86** | **57.14** | **78.57** | **71.43** | **64.29** | **64.29** |
